# Gait Cycle Duration Analysis in Lower Limb Amputees Using an IoT-Based Photonic Wearable Sensor: A Preliminary Proof-of-Concept Study

**DOI:** 10.3390/s25237148

**Published:** 2025-11-23

**Authors:** Bruna Alves, Alessandro Fantoni, José Pedro Matos, João Costa, Manuela Vieira

**Affiliations:** 1 Lisbon School of Engineering (ISEL), Polytechnic University of Lisbon (IPL), 1949-014 Lisboa, Portugal; bruna-alves@live.com.pt (B.A.); afantoni@deetc.isel.ipl.pt (A.F.); joao.costa@isel.pt (J.C.); 2 Escola Superior de Tecnologias da Saúde de Lisboa, Polytechnic University of Lisbon (IPL), 1990-096 Lisbon, Portugal; fulgenciomatos@estesl.ipl.pt; 3 Center of Technology and Systems (UNINOVA-CTS) and Associated Lab of Intelligent Systems (LASI), Quinta da Torre, Monte da Caparica, 2829-516 Caparica, Portugal; 4 DEE NOVA School of Science and Technology, New University of Lisbon, Quinta da Torre, Monte da Caparica, 2829-516 Caparica, Portugal

**Keywords:** gait symmetry, gait cycle duration, wearable sensor, LiDAR, prosthesis, lower limb amputation, IoT, rehabilitation, gait analysis

## Abstract

This study represents a preliminary proof of concept intended to demonstrate the feasibility of using a single-point LiDAR sensor for wearable gait analysis. The study presents a low-cost wearable sensor system that integrates a single-point LiDAR module and IoT connectivity to assess Gait Cycle Duration (GCD) and gait symmetry in real time. The device is positioned on the medial side of the calf to detect the contralateral limb crossing—used as a proxy for mid-stance—enabling the computation of GCD for both limbs and the derivation of the Symmetry Ratio and Symmetry Index. This was conducted under simulated walking at three cadences (slow, normal and fast). GCD estimated by the sensor was compared against the visual annotation with Kinovea^®^, showing reasonable agreement, with most cycle-wise relative differences below approximately 13% and both methods capturing similar symmetry trends. The wearable system operated reliably across different speeds, with an estimated materials cost of under 100 € and wireless data streaming to a cloud dashboard for real-time visualization. Although the validation is preliminary and limited to a single healthy participant and a video-based reference, the results support the feasibility of a photonic, IoT-based approach for portable and objective gait assessment, motivating future studies with larger and clinical cohorts and gold-standard references to quantify accuracy, repeatability and clinical utility.

## 1. Introduction

Lower limb amputation has a significant impact on individuals in quality of life, mobility and independence [1]. Gait recovery is the primary goal of rehabilitation for lower limb amputees (LLA), as locomotion capability is directly associated with quality of life [2]. In normal gait, there is a sequential and repetitive movement of the lower limbs, ensuring stable, symmetrical locomotion with minimal energy expenditure [3,4]. The loss of this capacity has physical, emotional, and social implications. So, humans try to maintain this ability even in the presence of severe impairments, such as amputation [2], often resulting in altered gait patterns [4]. In LLA, these changes are influenced by physiological and biomechanical factors that lead to instabilities, asymmetries, and increased energy expenditure [3]. The deviations are often compensatory gait deviations related either to individual impairments (joint contractures and muscle weakness) or prosthetic factors (misalignment, inappropriate component selection and poorly fitted socket) [5]. Identifying and correcting such deviations can reduce or prevent long-term health consequences and improve mobility and well-being [6].

Prescription and functional assessment of prosthetics are frequently based on empirical knowledge, with component selections criteria relying on subjective experience of clinicians, often disregarding individual functional needs [7]. This inadequacy can result in increased energy expenditure, joint pain, isolation, dependence on others for activities of daily living, and increased healthcare costs [5]. Thus, healthcare decisions should be evidence based, and prosthetic components should be prescribed in a scientifically grounded and justified manner [7,8]. Evidence based decisions require functional assessment tools that provide objective and quantitative data [5]. Quantitative measurement devices support clinical decisions for rehabilitation interventions based in scientific evidence [9].

Gait analysis is the primary method for functionally assessing lower limb prostheses and plays a crucial role in rehabilitation [10]. This set of procedures—used to observe, record, analyse, and interpret movement patterns—aims to obtain information that enhances performance and identifies gait abnormalities [11]. It enables healthcare professionals to identify gait disorders and supports prosthetic component selection and alignment adjustments. Proper identification of gait anomalies requires a comprehensive understanding of normal gait patterns [9].

The normal gait pattern results in a gait cycle with well-defined phases. The gait cycle is the interval between two consecutive occurrences of any repetitive gait event, which can be chosen to define the beginning of the cycle [12]. The gait cycle is divided into two phases, the stance phase and the swing phase, which correspond to the time that the foot is in contact with the ground, and in the air moving forward, respectively. These phases can be further divided into sub-phases: initial contact, loading response, mid-stance, heel-off, and push-off during stance, and acceleration, mid-swing, and deceleration during swing [13]. Throughout the gait cycle, parameters such as stride length, cadence, gait cycle duration, and gait symmetry can be evaluated [14]. Gait symmetry is associated with regularity, balance, good coordination, and reduced energy expenditure, making it an indicator of motor control and a criterion for tracking rehabilitation progress [15,16], and can be expressed through spatiotemporal ratios such as gait cycle duration [15]. Gait cycle duration is defined as the time interval between the occurrence of a specific gait phase and its next occurrence, representing the time required to complete one gait cycle [17]. To calculate this parameter, it is sufficient to identify a single specific event within the gait cycle and measure the time interval between its repetitions [18].

While some deviations from these parameters can be identified through observation, this qualitative assessment, though useful, depends on the observer’s subjective experience and lacks precision [10,19], other deviations must be measured and quantified using gait analysis devices capable of capturing three-dimensional motion and wearable sensors [20]. Quantitative methods record measurable kinetic, kinematic, and temporal parameters using devices of varying complexity [9]. These systems primarily rely on sensors, which respond to physical stimuli and transmit a resulting signal in the form of a measurement or control operation [21]. Advances in these devices have enabled the measurement of body locomotion and quantification of rehabilitation performance [22], allowing objective gait assessment and providing reliable data to professionals, thereby reducing the margin of error associated with subjective evaluations [19]. Many of these sensors are integrated into commercial clinical devices, typically expensive and used in laboratory environments without real-time analysis [23]. Others are integrated into wearable sensors, typically placed on various body segments or joints [19], with cost, size, and comfort during use being limiting factors [22].

Given the lack of accessible instruments capable of providing quantitative data for the objective assessment of lower limb amputees—while remaining both physically and economically viable in clinical contexts—a wearable sensor for detecting and analysing gait asymmetries based on Gait Cycle Duration was developed. The aim of this study is to apply the developed wearable sensor in assessing Gait Cycle Duration in transtibial amputees and to analyse gait symmetry through the Symmetry Index (SI) and Symmetry Ratio (SR). Additionally, the study aims to evaluate its potential as a clinical support tool, promoting evidence-based and sustainable rehabilitation adapted to the individual functional needs of users.

## 2. Related Work

Several solutions for assessing gait symmetry, particularly focusing on gait cycle duration, have been investigated, and quantitative gait analysis has been extensively studied using wearable and non-wearable sensing approaches, aiming to obtain reliable symmetry information outside controlled laboratory environments. Yang et al. developed a measurement device intended for lower limb amputees, consisting of an insole equipped with force-sensitive resistors, designed to be inserted into footwear. The insole is powered by a small battery located at the ankle, providing practical and non-invasive mobility for the user [23]. Nolan et al. used a similar system with the battery positioned at the waist. This system can measure, in real-time, the ground reaction forces generated by the feet during gait and based on this data determines the duration of the stance and swing phases to assess symmetry between the healthy and amputated limb [23,24]. Rathore et al. developed a device combining Force-Sensitive Resistors (FSRs) and potentiometers. The FSRs are placed at specific insole points to analyse plantar pressure distribution during the gait cycle, while the potentiometers measure joint angles at the knee and hip. This device calculates the gait cycle duration of the lower limbs by detecting gait phases corresponding to plantar pressure zones and recording the time interval between consecutive phases [20].

Other authors have used systems based on Inertial Measurement Units (IMUs). Maqbool et al. employed an IMU comprising a triaxial accelerometer and a triaxial gyroscope, positioned distally on the lower limbs to collect data on limb movement and positioning during gait. These data allowed the identification of different gait phases associated with acceleration and positioning, and subsequently the determination of gait cycle duration as well as stance and swing phase durations from two consecutive steps [25]. Steinmetzer et al. studied a similar device, equipped with a gyroscope, accelerometer, and magnetometer, which transmits collected data to a smartphone application. The sensors are placed on the distal segments of the lower limbs, and a convolutional neural network programmed to detect gait phases based on the relative positions of the limb segments determines the gait cycle duration [26].

Recent advances in wearable and non-wearable gait analysis have focused on integrating inertial sensors [25,26,27] and pressure-based indoles [20,23,25]. However, most of these systems require multiple sensors or laboratory environments, limiting portability. In contrast, the proposed LiDAR-based device enables detection of mid-stance with IoT connectivity, offering a low-cost and easily deployable alternative.

As observed in Table 1, previous systems typically rely on contact-based or multi-sensor setups, whereas the proposed LiDAR-IoT system provides a single point, non-contact solution capable of real-time gait symmetry assessment at low-cost.

## 3. Materials and Methods

### 3.1. Wearable Sensor

**Figure 1:**
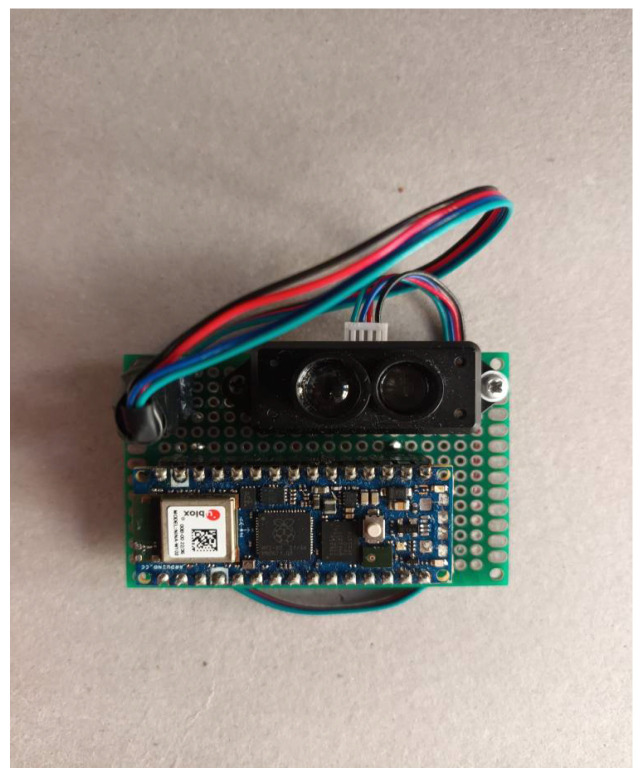
Wearable sensor designed.

Table 2 summarizes the main components and functionalities of the wearable system developed (Figure 1) for gait analysis, with particular emphasis on quantifying gait symmetry based on GCD. Using data acquired from the TF mini S LIDAR sensor, the system identifies the mid-stance phase—essential for determining the GCD. The algorithm then calculates both SI and SR: The wearable sensor integrates real-time processing and wireless connectivity, allowing seamless data transmission and interaction through a custom dashboard. The total estimated cost of the system is approximately 80 €, making it an affordable and accessible tool for gait analysis.

#### 3.1.1. TF Mini S LiDAR

The TFmini-S LiDAR by Benewake is a compact, single-point LiDAR module characterized by low power, small dimensions (42 mm × 15 mm × 16 mm), and a weight of approximately 5 g. The sensor has an operating range from 0.1 to 12 m, with a resolution of 1 cm and operates at an update rate of 1000 Hz, enabling high-speed data acquisition in real-time dynamic applications. The device exhibits ambient light immunity up to 70 Klux and supports an operating temperature range of 0 °C to 60 °C [27].

The sensor is powered by a 5 V supply, with an average current consumption of up to 140 mA and a power consumption of up to 0.7 W. It uses a VCSEL laser with a central wavelength of 850 nm and is classified as eye-safe (Class 1). The field of view (FOV) is approximately 2°. Communication with the sensor is achieved via UART or I^2^C interfaces, with adjustable baud rates of up to 400 kbps for I^2^C and 115,200 for UART [27].

In addition to the manufacturer’s specifications, experimental tests conducted validate the sensor’s suitability for detecting mid-stance during gait. Spectral analysis confirms a peak wavelength of 843.8 nm with a spectral width of approximately 1.1 nm, consistent with near-infrared emission and ensuring safe use in human environments. The TF mini-S demonstrated a sampling frequency of 3.987 kHz; this high acquisition rate is critical for accurate and continuous detection of mid-stance events across different walking speeds.

#### 3.1.2. Arduino Nano RP2040 Connect

The microcontroller used in the device was the Arduino^®^ Nano RP2040 Connect board (Torino, Italy), a platform that integrates the Raspberry Pi RP2040 microcontroller with advanced connectivity features and embedded sensors. This microcontroller is equipped with a 32-bit Dual-Core Arm^®^ Cortex^®^-M0+ processor operating at 133 MHz. It includes 264 kB of on-chip SRAM and supports up to 16 MB of external flash memory via QSPI interface. Additional features include a Direct Memory Access (DMA) controller, USB 1.1 support (host and device), and eight Programmable I/O (PIO) state machines for peripheral expansion [28].

Wi-Fi and Bluetooth connectivity are provided by the U-blox^®^ NINA-W102 module, which supports IEEE 802.11 b/g/n Wi-Fi in the 2.4 GHz band and Bluetooth 4.2. This module is powered by a 32-bit Dual-Core Xtensa LX6 processor running at 240 MHz, includes 520 kB of on-chip SRAM, and features an integrated PIFA antenna [28].

External memory is provided by a 16 MB NOR flash chip (AT25SF128A) with a QSPI data transfer rate of up to 532 Mbps and a program/erase cycle endurance of 100,000 cycles [28].

From a security perspective, the board integrates the Microchip^®^ ATECC608A cryptographic coprocessor, which provides secure key storage, support for symmetric algorithms (SHA-256, HMAC, AES-128), high-quality random number generation, and secure boot capabilities. Other features of the board include a common-cathode RGB LED controlled by the NINA-W102 module, 14 digital and 8 analog input/output pins with UART, SPI, and I^2^C communication support, and a Micro USB connector for power and programming. The onboard voltage regulator is a buck step-down converter, ensuring stable power delivery [28].

Electrically, the board can be powered via the ViN pin (4 V to 20 V) or through the USB connector (4.75 V to 5.25 V). The 3.3 V output provides up to 800 mA of current for user applications, including the onboard components [28].

#### 3.1.3. Arduino Cloud

The wearable sensor was programmed using the Arduino Cloud platform, which, in addition to enabling automatic storage of the generated data, allows real-time interaction and monitoring of the sensor. This platform supports Over-The-Air (OTA) communication with the Arduino Nano RP2040, eliminating the need for a continuous physical connection to the device during both development and operation. Furthermore, Arduino Cloud enabled the creation of a custom dashboard for controlling and monitoring the wearable sensor [29].

#### 3.1.4. Algorithm

To determine the Gait Cycle Duration, it is sufficient to identify a specific gait phase and measure the time interval between its consecutive occurrences [18]. In this study, the device was programmed to filter the data received from the LiDAR sensor to detect the mid-stance.

**Figure 2:**
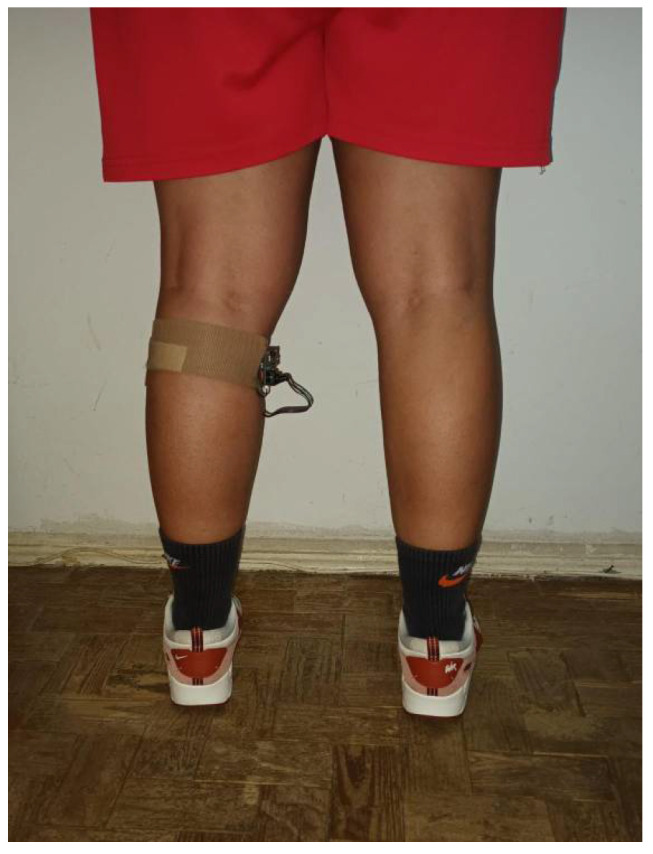
Rear view of the participant with the LiDAR device positioned on the left calf (posterior view).

The mid-stance coincides with the mid-swing in the contralateral limb, during which the limbs cross each other in the sagittal plane as gait progresses. To detect this limb-crossing event—used as a proxy for mid-stance—the LiDAR sensor integrated into the wearable sensor positioned to the medial side of the left gastrocnemius muscle, facing the contralateral limb to detect its crossing during walking. This configuration ensured a stable fixation and an unobstructed line of sight for the LiDAR beam (Figure 2).

**Figure 3:**
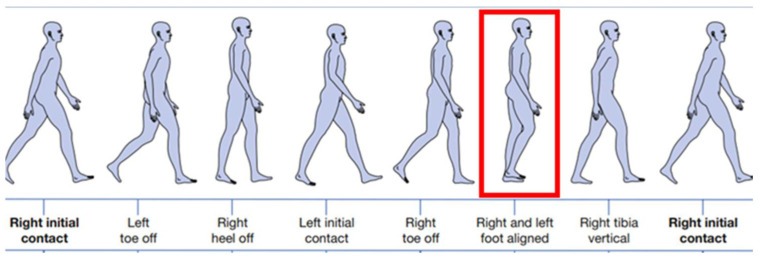
Gait Cycle. In bold the beginning and the end of the cycle. Mid-stance, detected by the sensor, is highlighted in the red rectangle.

To detect this crossing, the device was programmed to capture only distances up to 20 cm using the LiDAR sensor’s distance measurement mechanism. This threshold corresponds to the average step width in normal gait [14]. By restricting detection to this distance range, the device can identify the moment when the contralateral limb passes in front—thus marking the mid-stance (Figure 3) of the left leg. Within the same gait cycle, the mid-swing phase of the left leg occurs concurrently with the stance phase of the right leg [4], which is also detected by the sensor. As a result, the device is capable of simultaneously identifying the mid-stance of the left leg and the mid-swing of the right leg.

The time interval between consecutive events (detection of an object within 20 cm) does not correspond to a full gait cycle, but rather to a single step, as the sensor detects two events within the same cycle. Therefore, to accurately compute the Gait Cycle Duration for each limb, the device was programmed to measure the time interval between alternating events. This enables the simultaneous measurement of the complete Gait Cycle Duration for both the left and right lower limbs.

To calculate the SI and SR the algorithm is programmed to compute the average Gait Cycle Duration for each limb and to store the resulting data. Based on this information, the Symmetry Ratio is obtained through the direct ratio between the gait cycle durations of both limbs:

(1) $$SR=\;\frac{Tleft}{Tright}\;$$

The Symmetry Index is calculated using the normalized formula

(2) $$SI=\frac{Tleft-Tright}{0.5\times (Tleft+Tright)}\;$$

These two indicators are widely used to quantify gait symmetry due to their simplicity and ease of interpretation. The Symmetry Index is expressed as a percentage and is useful for quantifying the magnitude of asymmetry—where higher percentage values indicate greater gait asymmetry. The Symmetry Ratio, on the other hand, provides a direct comparison between the right and left limbs, reflecting their proportional relationship. A ratio of 1 indicates perfect symmetry, values greater than 1 indicate a longer gait cycle on the left limb, and values less than 1 indicate a longer gait cycle on the right limb [30].

#### 3.1.5. Dashboard

Through the Arduino Cloud platform, a Dashboard was developed to provide an intuitive and functional interface that enables real-time monitoring and control. This panel is directly integrated with the variables defined in the code, allowing dynamic visualization of the acquired data and immediate interaction with the system. The Dashboard was configured with specific widgets for each functionality, namely: control buttons (on/off and reset) that allow the device to be activated/deactivated and reset the measurement data; status indicators displaying whether the device is currently on or off; numerical displays for showing the gait cycle durations of both lower limbs, as well as the Symmetry Ratio and Symmetry Index; an elapsed time display showing the total evaluation duration in minutes; a dynamic graph visually representing the variation in gait cycles between the two limbs over time; and a notes field for recording observations during the evaluation.

The Dashboard can be accessed via the Arduino Cloud platform from either a computer or smartphone. When the device is active, it calculates the gait cycle durations based on the incoming data and updates the corresponding variables. When the start button is pressed, the system begins recording the gait cycle duration and calculates both the Symmetry Index and the Symmetry Ratio between the two limbs. The elapsed time is continuously updated, and the calculated values are sent to the platform and displayed on the dashboard.

**Figure 4:**
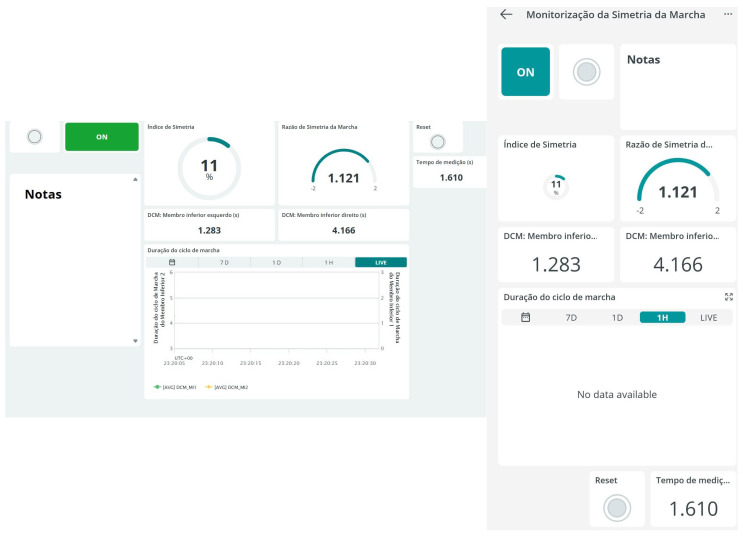
Dashboard-web and app versions.

The Dashboard also allows the user to interact with the device, including pausing or resetting the measurement using the widgets. Pressing the reset button clears all recorded values, enabling a new evaluation to be initiated. Data can be viewed in real time based on the information transmitted by the device, facilitating continuous monitoring of the patient’s gait, see Figure 4.

#### 3.1.6. Data Processing

To simplify result interpretation and facilitate later consultation, a simple web interface was developed. This web interface was designed to ease the upload of CSV files from the Arduino Cloud, process the contained data, and automatically generate a PDF report that compiles information on the Gait Cycle Duration of both limbs, the Symmetry Index, the Symmetry Ratio, a graph illustrating the variation in Gait Cycle Duration for each limb, and a section for adding evaluation notes.

The application was implemented in Python 3.10.7, using the Flask framework to create the web application that provides the page for CSV file uploads and a notes input field. Data handling was carried out using the Pandas library for reading, converting, and merging the data from the CSV files. The graphs were created using the Matplotlib 3.10.7 library and embedded into the PDF report. The PDF report itself was generated with the FPDF library and compiles the title and data description using tables and graphs derived from the CSV data.

The application was programmed to include on the first page of the final report the title “Gait Symmetry Report” and the subtitle “Gait Cycle Duration”, followed by a table summarizing the mean values, the symmetry index, and the symmetry ratio. A grouped bar chart comparing gait cycle durations between the two limbs is then generated, saved as an image in a temporary directory, and inserted into the PDF. For each CSV file, a dedicated page is created containing a table with two columns: the timestamp and the corresponding gait cycle duration in seconds, both formatted to allow for a clear and systematic reading of the data. When the user submits notes through the web interface form, these are added to the final page of the PDF, enabling contextualization of the results and the inclusion of any additional comments related to the analysis. This procedure allows for detailed analysis of the data, facilitating storage, interpretation, and longitudinal follow-up.

### 3.2. Methodology

The wearable sensor, integrating the TF mini S LiDAR and the Arduino Nano RP2040, was positioned on the medial side of the left, specifically at the level of the gastrocnemius muscle belly. The choice of the left leg was made solely for consistency across all tests and for practical reasons related to sensor alignment. As the LiDAR beam captures the crossing of the contralateral limb, the selected side does not influence the results, ensuring equivalent measurements if the device were mounted on the opposite leg. Mounting the device on the left calf ensured a stable attachment point and a clear line of sight toward the opposite leg,

To evaluate the device’s ability to capture different walking conditions, three gait simulations were performed to simulate fast gait, normal gait and slow gait resulting in different GCD. The data were transmitted and visualized in real time using the Dashboard and collected using the method described in Section 3.1.6.

**Figure 5:**
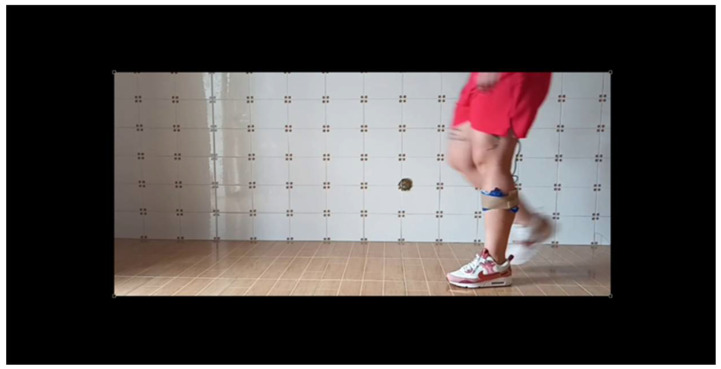
First mid-stance event detected from video analysis.

**Figure 6:**
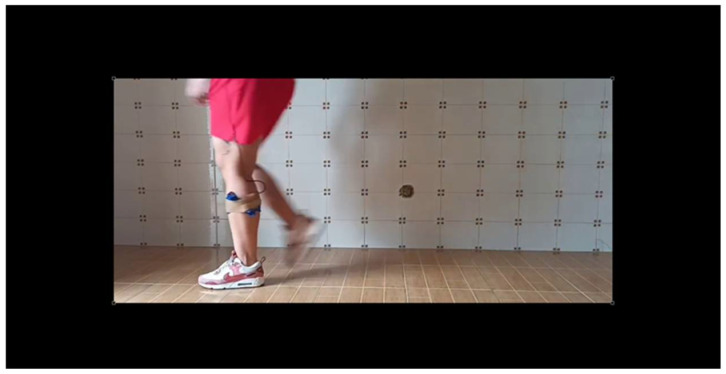
Second mid-stance event of the same gait cycle.

To assess the system’s accuracy, an additional test was conducted using Kinovea^®^ Version: 0.9.5, a motion analysis software [31]. A video of the subject walking with the sensor was recorded while simultaneously capturing data from the wearable device. This allows us to align the visual data with the device readings. The procedure was as follows:

1. Video Recording: The subject was filmed walking while wearing the device, using a standard camera placed in the sagittal plane.
2. Visual detection: Using Kinovea^®^, two consecutive mid-stance events were manually marked for both left (Figure 5) and right (Figure 6) limbs across four gait cycles.
3. Time extraction: The time stamps of these events were used to calculate the GDC by measuring the time intervals between repeated mid-stance events.
4. Comparison: The GCD values obtained from the wearable sensor were compared to those derived from the Kinovea^®^ analysis. For each cycle, the absolute and the relative differences were calculated.

This approach enables a preliminary assessment of the accuracy of the wearable device in detecting gait events in comparison to a conventional method.

Figure 5 and Figure 6 illustrate the two consecutive mid-stance instants identified from the Kinovea^®^ video frames, corresponding respectively to the supporting phases of the left and right legs. The time interval between these two events represents one complete gait cycle and was used to calculate the Gait Cycle Duration (GCD). This visual annotation was adopted as the reference for comparison with the LiDAR-based detection.

### 3.3. Experimental Setup

The experiments were carried out indoors, on a flat and unobstructed surface under standard lighting conditions (Table 3). A single healthy adult volunteer (female, 31 years old, 1.61 m, 60 kg) participated in the preliminary validation. Three walking trials were recorded at slow, normal, and fast cadences, each lasting approximately 10 s and including at least four gait cycles.

The LiDAR sensor was attached to the medial side of the left calf, facing the contralateral limb to detect crossing events associated with mid-stance instants. The recorded LiDAR distance signals were synchronized with a reference video captured at 30 fps using a smartphone camera. Gait events were annotated manually in Kinovea^®^ software to obtain the visual reference for comparison.

The time interval between consecutive mid-stance instants in the reference video was considered the ground-truth Gait Cycle Duration (GCD). The relative difference between the GCD obtained from the LiDAR signal and the one annotated in the video was used to evaluate the accuracy of the proposed system.

## 4. Results

### 4.1. Gait Cycle Duration Across Different Speeds

The sensor successfully detected GDC under different gait conditions, showing sensitivity to variations in walking speed. As shown in Table 4, GCD decreased with increasing gait speed, as expected. The sensor captured short, normal and long GCD, confirming the sensor’s ability to track cadence variation effectively.

### 4.2. Comparison with Kinovea

To validate the sensor’s accuracy, a cycle-by-cycle comparison was performed against Kinovea^®^. For the left leg (Table 5), the absolute differences ranged from 0.07 s to 0.26 s, with relative differences between 4.73% and 17.22%. For the right leg (Table 6), absolute differences varied from 0.01 s to 0.43 s, corresponding to relative differences of 0.71% to 29.66%.

Mean GDC values and symmetry metrics are summarized in Table 7. The calculated SI and SR indicate that both systems detect a relatively symmetric gait, although the sensor showed a higher symmetry Index (5.05%) compared to Kinovea^®^ (0.52%).

## 5. Discussion

The LiDAR-based gait event detection demonstrated consistent operation across the three cadences tested. The system successfully identified mid-stance instants from the LiDAR sensor and computed Gait Cycle Duration (GCD) values comparable to those obtained from the Kinovea^®^ visual annotation. Although small variations were observed between trials, both approaches captured the same symmetry trend—slightly longer GCD for the left limb—confirming that the sensor accurately detects alternating gait events.

The main sources of error are associated with the dynamic behavior of the sensor during motion, the manual identification of reference events in the video, and the intrinsic resolution of each measurement system. The video reference was recorded at 30 fps, corresponding to a temporal resolution of approximately 33 ms per frame, which may lead to small timing differences when compared with the LiDAR output.

The observed differences between the left and right leg results are minor and arise primarily from measurement-related factors rather than true gait asymmetry. Because the LiDAR device was fixed on the left leg, detection of the contralateral (right) limb occurred while the sensor itself was moving, which can slightly influence the temporal precision of distance readings. Additional sources of variation include the manual identification of mid-stance events in Kinovea^®^ and the 30 fps frame-rate of the video reference (≈33 ms temporal resolution). In contrast, the TFmini-S operated at an effective sampling rate of approximately 3.987 kHz, providing much finer temporal resolution; hence, the discrepancies observed fall within expected experimental variability and measurement precision. Similar inter-limb variations have been reported in optical and inertial gait analysis of healthy subjects, confirming that the present results reflect normal variability rather than systematic bias. Despite these factors, both methods revealed consistent gait-cycle trends across cadences, confirming that the proposed LiDAR-based approach reliably captures the alternating limb pattern and overall symmetry of gait.

The results confirm the feasibility of using a compact photonic sensor to estimate gait parameters in real time, offering a portable and low-cost alternative to camera-based or inertial systems for preliminary gait assessment.

### Limitations and Future Work

While the results presented offer promising evidence of the sensor’s ability to detect mid-stance and determine gait cycle duration, some limitations must be acknowledged. The current validation was conducted with a single healthy participant, which restricts the generalizability of the results. The system was tested only on one limb, with gait symmetry inferred indirectly from consecutive detections of the contralateral limb, which may introduce minor asymmetry-related variations. In addition, all experiments were carried out in a controlled indoor environment and compared against a visual reference method (Kinovea^®^), which is subject to frame-selection subjectivity and limited temporal precision.

The validation performed in this study involved a single healthy participant, and therefore the results must be interpreted strictly as proof-of-concept. Future work will include testing with a larger sample (≥10 participants) to properly assess repeatability, variability and generalization. Future validation should include a larger and more diverse sample, particularly individuals with pathological gait, such as lower limb amputees using prostheses. Testing the device on both limbs alternately is also recommended to assess consistency and identify any side-related variability in detection. Moreover, more robust and accurate reference systems—such as inertial measurement units or pressure platforms—should be employed as gold-standard methods to validate the proposed wearable sensor. These tools enable more precise gait event detection and minimize the influence of subjective interpretation. Extended walking trials with continuous gait cycles and the application of statistical methods will also be necessary to quantify the reliability of the system and support its potential for clinical application.

## 6. Conclusions

This study presented the development and preliminary evaluation of a wearable sensor system integrating LiDAR and IoT technologies for the assessment of Gait cycle Duration and gait symmetry in lower limb amputees. The device demonstrated the ability to detect mid-stance and calculate GDC across a range of walking speeds.

The comparative analysis with Kinovea^®^ confirmed a reasonable agreement between the wearable system and the visual annotation method, particularly in identifying symmetrical patterns and gait cycle consistency. Although some discrepancies were observed—these can be attributed to the limitation of video-based analysis, sensor displacement during gait, and the reduced number of samples.

The findings support the device’s feasibility as a portable, real-time, gait analysis tool, with potential applications in prosthetic fitting and rehabilitation monitoring. Its low cost, ease of use, and integration with cloud-based platforms position it as a promising solution for more accessible and evidence-based clinical decision-making.

However, further validation is essential Future studies involve a broader and more diverse participant base, employ gold-standard reference systems, and apply statistical methods to evaluate measurement accuracy and repeatability.

## Figures and Tables

**Table 1:** Comparison of representative gait-analysis devices and the proposed LiDAR–IoT system.

Study	Sensor Type	Participants	Main Goal/Method	Limitations
Yang et al. (2012) [23]	In-shoe FSR sensors with wireless feedback (LEAFS)	3 transtibial amputees	Real-time auditory feedback to reduce stance-time asymmetry	Very small sample; contact sensors; lab-supervised training
Sant’Anna et al. (2013) [22]	IMU (accelerometer + gyroscope) at waist and shanks	19 healthy + 11 hip-replacement patients	Quantify gait symmetry/normality; compared against 3D motion capture	Multiple sensors; setup complexity
Nolan et al. (2003) [24]	Instrumented force-shoe system (vGRF at 50 Hz)	4 TF + 4 TT amputees; 6 able-bodied	Assess effects of walking speed on temporal/loading asymmetry	Bulky footwear instrumentation; no real-time feedback
Maqbool et al. (2024) [25]	Dual IMUs on both shanks (+FSR validation)	7 controls + 1 TFA + 1 TTA	Evaluate asymmetry across inner stance/swing phases (OG & TM)	Limited amputee sample; IMU-only approach
This work	Single-point LiDAR	1 healthy (pilot)	Detect mid-stance events → compute GCD, Symmetry Ratio/Index	Preliminary validation; single participant; video-based reference

**Table 2:** Wearable Sensor Composition.

Component	Function
TF mini S LiDAR	Detection of the contralateral limb during gait from the sagittal plane—corresponds to mid-stance detection.
Arduino Nano RP2040	Real-time data processing, Wi-Fi connectivity, and integration with Arduino Cloud.
Arduino Cloud	Data storage, device configuration, and programming interface.
Algorithm	Calculation of Gait Cycle Duration and determination of Symmetry Index, and Symmetry Ratio.
Dashboard	Real-time data access and user interaction with the wearable sensor via smartphone or computer.

**Table 3:** Summary of experimental conditions.

Parameter	Description
Participant	1 healthy adult
Number of trials	3 (slow, normal, fast cadences)
Duration per trial	10 s
Surface	Indoor, flat
Reference method	Visual annotation using Kinovea^®^

**Table 4:** GCD across different speeds.

	Left Leg GCD (s)	Right Leg GCD (s)
Fast gait	0.615	0.619
Normal gait	1.080	1.073
Slow gait	2.398	2.429

**Table 5:** Cycle-by-cycle comparison—Left leg.

Cycle	Kinovea (s)	Sensor (s)	Abs. Diff. (s)	Rel. Diff. (%)
1	1.38	1.31	0.07	5.07%
2	1.48	1.55	0.07	4.73%
3	1.51	1.25	0.26	17.22%
4	1.41	1.58	0.17	12.06%

**Table 6:** Cycle-by-cycle comparison—Right leg.

Cycle	Kinovea (s)	Sensor (s)	Abs. Diff. (s)	Rel. Diff. (%)
1	1.41	1.40	0.01	0.71%
2	1.51	1.78	0.27	17.88%
3	1.45	1.02	0.43	29.66%
4	1.38	1.21	0.17	12.32%

**Table 7:** Mean values and symmetry indices.

	Left Leg GCD (s)	Right Leg GCD (s)	Symmetry Index (%)	Symmetry Ratio
Kinovea	1.445	1.437	0.52%	1.005
Sensor	1.423	1.353	5.05%	1.052

## Data Availability

Dataset available on request from the authors.

## Abbreviations

GCD	Gait Cycle Duration
LLA	Lower Limb Amputee
FSRs	Force-Sensitive Resistors
IMU	Inertial Measurement Units
